# First Insights into the Genome of the Moderately Thermophilic Bacterium *Clostridium tepidiprofundi* SG 508^T^

**DOI:** 10.1128/genomeA.00379-16

**Published:** 2016-05-12

**Authors:** Anja Poehlein, Ines Friedrich, Larissa Krüger, Rolf Daniel

**Affiliations:** a Genomic and Applied Microbiology & Göttingen Genomics Laboratory, Institute of Microbiology and Genetics, Georg-August University, Göttingen, Germany; b Members of the Applied Bioinformatics in Microbiology Course of the Microbiology and Biochemistry MSc/PhD program, Georg-August University, Göttingen, Germany

## Abstract

The moderately thermophilic bacterium *Clostridium tepidiprofundi* is Gram-positive and belongs to clostridial cluster I. It was isolated from a hydrothermal vent chimney. Substrates utilized by *C. tepidiprofundi* include casein, peptone, tryptone, yeast extract, beef extract, starch, maltose, and glucose. The genome consists of one replicon (3.06 Mb).

## GENOME ANNOUNCEMENT

The anaerobic, moderately thermophilic, fermentative bacterium *Clostridium tepidiprofundi* is a Gram-positive bacterium with a straight to slightly curved rod shape (1). The *C. tepidiprofundi* type strain SG 508^T^ was isolated from a hydrothermal vent chimney located at 13°N on the East Pacific Rise at a depth of 2,650 m (1). This strain is able to gain energy by degrading amino acids via Stickland reaction (2). The cells are 0.4 to 0.6 µm in diameter and 2.0 to 3.0 µm in length (1). The growth temperature ranges from 22 to 60°C. The substrates that are utilized include casein, peptone, tryptone, yeast extract, beef extract, starch, maltose, and glucose (1). *C. tepidiprofundi* belongs to cluster I of clostridia.

Chromosomal DNA of *C. tepidiprofundi* SG 508^T^ was isolated using the MasterPure complete DNA purification kit (Epicentre, Madison, WI, USA). The extracted DNA was used to generate Illumina shotgun sequencing libraries according to the manufacturer’s protocol (Illumina, San Diego, CA, USA). Sequencing was conducted using a MiSeq instrument and MiSeq reagent kit version 3 as recommended by the manufacturer (Illumina). Sequencing resulted in 1,493,516 paired-end reads that were trimmed using Trimmomatic version 0.32 (3). Genome sequence assembly with SPAdes version 3.6.2 (4) resulted in 175 contigs (>500 bp) and an average coverage of 105.65-fold. For the validation of the assembly, QualiMap version 2.1 was used (5). The size of the draft genome and the GC content are 3.06 Mb and 29.44 %, respectively. The software tool Prokka (6) was used for automatic gene prediction and automatic annotation. The draft genome contains 10 rRNA genes, 92 tRNA genes, and 1,956 predicted protein-encoding genes with function prediction and 773 genes encoding hypothetical proteins. The genome of *C. tepidiprofundi* contains all genes coding for proteins necessary for glycine, sarcosine, and betaine reduction. We identified one gene cluster encoding glycine and one encoding betaine reductase. The putative glycine reductase complex-encoding operon (*grdx-trxBA-grdEABCD*) of *C. tepidiprofundi* showed an organization, which is similar to that of the corresponding operons of *C. aceticum* (7), *Eubacterium acidaminophilum* (8), and *Sporomusa ovata* (9). In accordance with its lifestyle, *C. tepidiprofundi* harbors a potential gene encoding a thermostable DNA polymerase. Genome analysis indicated that *C. tepidiprofundi* produces solvents like ethanol and butanol. Genes encoding all key genes for ethanol production, such as aldehyde dehydrogenase and two bifunctional aldehyde-alcohol dehydrogenases, were detected. The putative genes for key enzymes of butanol formation comprised butyryl-CoA dehydrogenase, acetyl-CoA acetyltransferase, 3-hydroxybutyryl-CoA dehydrogenase, 3-hydroxybutyryl-CoA dehydratase, several alcohol dehydrogenases, acetate kinase, phosphate acetyltransferase, butyrate kinase, and phosphate butyryltransferase.

### Nucleotide sequence accession numbers.

The whole-genome shotgun project has been deposited at DDBJ/EMBL/GenBank under the accession number LTBA00000000. The version described here is the first version, LTBA01000000.
